# Burden of infectious diseases in prison settings and services offered

**DOI:** 10.1093/eurpub/ckac129.385

**Published:** 2022-10-25

**Authors:** F Alves da Costa

**Affiliations:** WHO Regional Office for Europe, Copenhagen, Denmark

## Abstract

**Introduction:**

The WHO Prison Health Framework was developed to assess prison health system performance and support Member States (MS) in improving their prison health systems. Moreover, it shall enhance MS capacity to evaluate: the impact of changes in governance models, progress in service provision and improvements of the health status of people in prison (PiP).

**Methods:**

The framework informed the 2021 data collection round of the Health In Prisons European Database Survey. Invitations were sent to all 53 MS of the WHO European Region. Those MS nominating a focal point and providing valid answers were included in the analysis.

**Results:**

Answers were obtained from 36 MS, representing a total of 613,497 PiP. Access to immunization was very good across all MS, with the highest for COVID-19 (90% of MS provide it in all prisons). Vaccination against HBV was only available in all prisons of 25 MS. Access in all prisons to HIV post and PrEP were reported, respectively, by 78% and 58% of MS. Screening for diseases at entrance was common for HIV, HCV and HBV. In all prisons of 35 MS soap was provided for free, while needles & syringes and lubricants were only provided free of charge, respectively, in 3 and 4 MS. 5 MS did not have therapeutic spaces to tackle drug problems in any prison, in 73% of those having, accessibility was restricted to some prisons. HIV prevalence ranged from 0-16% and treatment was accessible to 55-100% of those diagnosed. Prevalence of HCV ranged from 0-34%, with access to treatment ranging from 0-91%. The most common format of health records in European prisons was paper based (44%).

**Conclusions:**

Prison-based data collection systems resulted in limited capacity for extraction so that some countries were unable to provide any data on disease prevalence or treatments offered. Given the scarcity of data on this topic obtained from real-world and not from ad-hoc studies, this snapshot provides an important contribution to public health.

